# The cost of living in larger primate groups includes higher fly densities

**DOI:** 10.1007/s10393-022-01597-5

**Published:** 2022-06-04

**Authors:** Jan F. Gogarten, Mueena Jahan, Sébastien Calvignac-Spencer, Colin A. Chapman, Tony L. Goldberg, Fabian H. Leendertz, Jessica M. Rothman

**Affiliations:** 1 Epidemiology of Highly Pathogenic Organisms, Robert Koch Institute, Berlin, Germany; 2 Viral Evolution, Robert Koch Institute, Berlin, Germany; 3 Applied Zoology and Nature Conservation, University of Greifswald, Greifswald, Germany; 4 Department of Microbiology and Public Health, Bangabandhu Sheikh Mujibur Rahman Agricultural University, Gazipur, Bangladesh; 5 Center for the Advanced Study of Human Paleobiology, George Washington University, Washington, DC USA; 6 School of Life Sciences, University of KwaZulu-Natal, Pietermaritzburg, South Africa; 7 Shaanxi Key Laboratory for Animal Conservation, Northwest University, Xi’an, China; 8 Department of Pathobiological Sciences, University of Wisconsin - Madison, Madison, WI USA; 9 Helmholtz Institute for One Health, Greifswald, Germany; 10 Department of Anthropology, Hunter College, City University of New York, New York, NY USA

**Keywords:** disease risk, disease vector, non-human primates, sociality and health

## Abstract

Flies are implicated in carrying and mechanically transmitting many primate pathogens. We investigated how fly associations vary across six monkey species (*Cercopithecus ascanius, Cercopithecus mitis*, *Colobus guereza*, *Lophocebus albigena*, *Papio anubis*, and *Piliocolobus tephrosceles*) and whether monkey group size impacts fly densities. Fly densities were generally higher inside groups than outside them, and considering data from these primate species together revealed that larger groups harbored more flies. Within species, this pattern was strongest for colobine monkeys, and we speculate this might be due to their smaller home ranges, suggesting that movement patterns may influence fly–primate associations. Fly associations increase with group sizes and may thus represent a cost to sociality.

**Supplementary Information:**

The online version contains supplementary material available at 10.1007/s10393-022-01597-5.

## Introduction

The order Primates contains many extremely social members, with over two-thirds of species forming permanent year-round groups. Group living may increase disease risk through the attraction of more arthropod vectors (van Schaik and Kappeler 1997); for example, increasing group sizes are associated with increased malaria infection rates across species of platyrrhine primates (Davies et al. 1991; Nunn and Heymann 2005). Conversely, disease control and avoidance are hypothesized to influence the size and behavior of primate groups (Freeland 1976); group living may reduce between-group contacts and limit disease transmission at the population level (Freeland 1976; Manlove et al. 2014), while increased modularity of social networks may mediate the higher disease risk associated with living in larger groups (Griffin and Nunn 2012).

Research on primate sociality and disease vectors has predominantly targeted biological vectors, i.e., those in which infectious agents must replicate to complete parts of a life cycle (e.g., malaria parasites in mosquitos). Much less consideration has been given to mechanical vectors, such as flies. Synanthropic flies associated with human settlements and their livestock can transmit a diversity of important pathogens mechanically, including bacteria (e.g., *Chlamydia trachomatis* (Forsey and Darougar 1981)), protozoan parasites (e.g., *Cryptosporidium parvum* (Clavel et al. 2002)), helminth eggs (e.g., *Ascaris lumbricoides* (Adenusi and Adewoga 2013)), and viruses (e.g., turkey coronavirus (Calibeo-Hayes et al. 2003)). Higher fly densities are associated with increased human disease risk (Graczyk et al. 2001), though it is unclear whether fly densities vary with human population densities or the size of groups.

Fly associations are not unique to human environments. Terrestrial groups of sooty mangabeys (*Cercocebus atys*) and chimpanzees (*Pan troglodytes*), in Taï National Park, Côte d’Ivoire harbored higher fly densities inside than outside social groups (Gogarten et al. 2019). Individual flies followed a mangabey group for up to 13 days, suggesting a stable association (Gogarten et al. 2019). Furthermore, these flies carried viable *Bacillus cereus* biovar *anthracis*, which causes sylvatic anthrax (Hoffmann et al. 2017; Gogarten et al. 2019), suggesting flies pose a significant disease risk. For wild primates, it is also unclear how fly densities vary with group sizes.

To assess the generality of stable fly–primate associations, as well as to investigate host factors that might influence these associations, we examine the effect of group size on fly densities in six sympatric, arboreal primate species in Kibale National Park, Uganda.

## Methods

**Figure 1:**
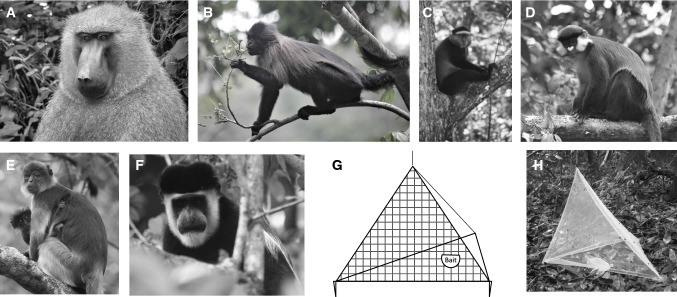
Fly densities were measured inside and outside groups of **A** Olive baboons, **B** Gray-cheeked mangabeys, **C** Blue guenons, **D** Red-tailed guenons, **E** Red colobus, and **F** Black-and-white colobus using **G** + **H** Custom-made traps made of mesh.

Kibale National Park, Uganda (0°13′–0°41’N and 30°19′–30°32′E), contains 13 species of non-human primates from the families Hominidae, Cercopithecidae, Galagidae, and Lorisidae, of which we studied six species from the family Cercopithecidae species (Fig. 1A–F): black-and-white colobus (*Colobus guereza*), blue guenons (*Cercopithecus mitis*), gray-cheeked mangabeys (*Lophocebus albigena*), olive baboons (*Papio anubis*), red colobus (*Piliocolobus tephrosceles*), and red-tailed guenons (*Cercopithecus ascanius*). These species are largely arboreal, with the exception of olive baboons, which spend much of their day on the ground. Many groups are habituated to human observers, so they can be easily approached and studied (Gogarten et al. 2015).

Flies were captured using custom-made traps (described in: Hoffmann et al. 2017) placed over a commercial attractant based on animal proteins that mimics scent emitted by a decaying carcass (Unkonventionelle Produkte Feldner, Waldsee, Germany; Fig. 1 G and H). Following Gogarten et al., (2019), we controlled for location and temporal variation in fly densities by setting pairs of traps, one for 20 min roughly at the center of a group (estimated visually; hereafter referred to as ‘in group’) and subsequently walked 500 m from the trap location in the group to set a second trap for 20 min (hereafter referred to as ‘away from the group’). Traps were set in and away from groups of black-and-white colobus (*N*_paired traps_ = 50; *N*_groups_ = 31), blue guenons (*N*_paired traps_ = 22; *N*_groups_ = 11), gray-cheeked mangabeys (*N*_paired traps_ = 32; *N*_groups_ = 17), olive baboons (*N*_paired traps_ = 23; *N*_groups_ = 9), red colobus (*N*_paired traps_ = 24; *N*_groups_ = 20), and red-tailed guenons (*N*_paired traps_ = 25; *N*_groups_ = 20). For habituated groups, group sizes were available from long-term studies (Gogarten et al. 2015). For unhabituated groups, we estimated group sizes by waiting until a group made a movement across a canopy opening (e.g., a treefall gap or forest path) and counted individuals as they passed. Sample sizes were determined by group encounter rates.

To test the hypothesis that fly densities were higher inside than outside groups, we conducted separate paired one-tailed t-tests comparing fly densities inside and outside groups of each primate species. We log-transformed fly density estimates and present back transformed means. Due to small sample sizes for many of the cercopithecine species, we also performed a *t* test combining data from the cercopithecine monkeys. To examine a potential relationship between group size and fly density, we calculated the primate-associated excess fly density for each trap by taking the difference from the paired control trap. For each group, we then calculated the average excess density of flies across all traps. We used a linear regression to test for a relationship between the average excess fly density and group size: once for all primate groups combined and then separately for each species. Averaging fly densities for groups with multiple fly density estimates avoided introduction of biases stemming from the use of repeated measures in linear models. While such averaging represents a loss of information about within-group variation that might be incorporated in a generalized linear mixed model (GLMM) framework, in this case most groups were sampled only once (*N* = 92 of the 108 sampled), which precluded the reliable estimation of a within-group random effects in a GLMM, so this modeling approach was not used here.

To determine the fly species present, we used soup metabarcoding of a fragment of the mitochondrial gene, cytochrome oxidase C subunit 1 (COI), with the ‘ANML’ primers adapted with an Illumina adapter (Jusino et al. 2019). A leg was removed from 100 flies captured in groups of each primate species (with the exception of red-tailed guenons, for which 75 flies were available), for a total of 575 fly legs (Table S1). Fly legs were pooled by primate species and homogenized with a Fast Prep (MP Biomedicals). To explore whether the same fly species were present outside primate groups, we homogenized fly legs from the same number of flies captured outside groups (*N*_flies_ = 575, *N*_pools_ = 6). DNA was extracted with the GeneMATRIX Stool DNA Purification Kit (Roboklon). A pool of 100 fly legs from flies captured in the Volkspark Rehberge, Berlin, Germany, and an extraction blank were included as controls. Duplicate PCR amplification reactions for each sample and control were carried out in a volume of 15 µl, with 0.2 mM dNTP, 4 mM MgCl2, 0.2 µM of each primer, 1.25U Platinum® Taq polymerase (Invitrogen), 2.5 µl 10 × PCR buffer (Invitrogen), and PCR water. Reactions were seeded with 200 ng DNA extract. Three negative controls were included with the PCR. Cycling conditions followed Jusino et al. (2019). Products were visualized on agarose gels and cleaned using AMPure XP Beads and pools uniquely dual indexed using the Nextera XT Index kit and sequenced on an Illumina NextSeq 500 with a mid-output kit v.2 and 2 × 150 cycles.

Primers were removed with cutadapt v2.1 and sequences cleaned of adapters and trimmed for quality with Trimmomatic v0.38, removing the leading and trailing bases below Q30, and clipping any part of the read where the average base quality across 4 bp was less than 30. Poor read quality for the second read precluded its use in the analysis. Reads were assigned to taxa using the eukaryote CO1 reference set v4.0 with the RDP classifier (Wang et al. 2007; Porter and Hajibabaei 2018). A bootstrap support cutoff value of 0.6 was selected, as this was shown to produce at least 99% correct assignments with barcodes of this length (Porter and Hajibabaei 2018). Negative controls did not include reads assigned to the family Diptera, but the extraction blank included reads assigned to taxa found in Berlin. To account for this, for each sample, we excluded taxa with less than two times the absolute number of reads detected in the extraction blanks that simultaneously represented less than 1 / (2 × *N*_fly legs in the pool_) of the reads for that pool. In addition, to reduce the risk of artifacts we considered taxa present in a sample only when more than 10 reads were assigned to it.

To examine differences in fly species community composition by primate species and within or outside a group, we calculated the Bray–Curtis dissimilarity of the species-level community matrix among all the Kibale fly pools and clustered communities using the agglomerative hierarchical clustering algorithm with the unweighted pair group method with arithmetic mean (UPGMA) method. To formally test for differences in fly community composition between flies captured within or outside the group, we used the adonis function in the *vegan* R package v2.5–7, which calculates an ANOVA-like test of the variance in beta diversity explained by categorical variables (Oksanen et al. 2020). Statistical analyses were performed in R v4.1.1 (R Core Team 2021).

## Results

**Figure 2:**
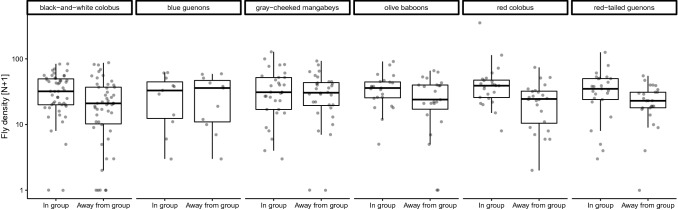
Fly densities within and outside primate groups for six different species in Kibale National Park, Uganda. The middle horizontal line represents the median, while the rectangle shows the quartiles and the vertical line represents the 2.5 and 97.5% percentiles, and each circle indicates the number of flies caught in a particular trap on a particular day.

Fly density was significantly higher inside groups of the folivorous black-and-white colobus and red colobus (Fig. 2; Table 1). Fly densities were also higher inside groups of frugivorous olive baboons, but not inside groups of blue guenons, gray-cheeked mangabeys, or red-tail guenons (Fig. 2; Table 1), though when combining data from all cercopithecine species, we also found significantly more flies inside groups ($\bar{x}_{{{\text{in}}\,\, {\text{group}}}}  = 36.3$, $\bar{x}$_away from group_ = 28.8, *t* = 2.491, d*f* = 90, *P* < 0.01). The highest fly densities inside groups were observed for red colobus monkeys and the lowest for blue guenons (Table 1).

**Table 1:** Comparison of fly densities inside and outside groups of monkeys.

Species	N flies		Paired *t* test		
	$\bar{x}$_in group_	$\bar{x}$_away from group_	*t*	d*f*	*P*
Black-and-white colobus *Colobus guereza*	34.3	25.4	3.630	49	< **0.001**
Blue guenons *Cercopithecus mitis*	30.5	29.5	0.266	10	0.398
Gray-cheeked mangabey *Lophocebus albigena*	37.8	32.8	1.089	31	0.148
Olive baboons *Papio anubis*	36.4	28.3	1.731	22	**0.046**
Red colobus *Piliocolobus tephrosceles*	51.7	24.4	3.038	23	< **0.005**
Red-tailed guenons *Cercopithecus ascanius*	36.8	23.7	1.577	24	0.064

Bold values indicates Significant values

**Figure 3:**
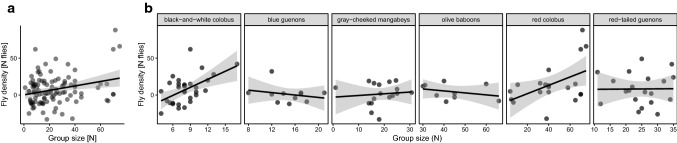
Relationships between group sizes and average fly densities. The graphs display fly densities and group sizes for **A** All primate species combined and **B** Different primate groups within each primate species. Each point represents the average value for a particular social group. Solid lines represent the least-squares regression lines, and gray shading indicates 95% confidence intervals.

Fly density increased with group size when groups of all species were considered together (R^2^ = 0.064, F_1,106_ = 8.325, *P* < 0.005; Fig. 3A). When examining within-species relationships, the strongest relationship between group size and fly density was observed for black-and-white colobus (R^2^ = 0.253, F_1,29_ = 11.180, *P* < 0.005) and red colobus (R^2^ = 0.188, F_1,18_ = 5.407, *P* = 0.032), whereas no relationship was observed in blue guenons (Adjusted R^2^ = – 0.044, F_1,9_ = 0.582, *P* = 0.465), gray-cheeked mangabeys (adjusted R^2^ = – 0.056, F_1,15_ = 0.156, *P* = 0.70), olive baboons (adjusted R^2^ = – 0.033, F_1,7_ = 0.747, *P* = 0.42), or red-tail guenons (adjusted R^2^ = – 0.055, F_1,18_ = 0.0019, *P* = 0.97; Fig. 3B).

**Figure 4:**
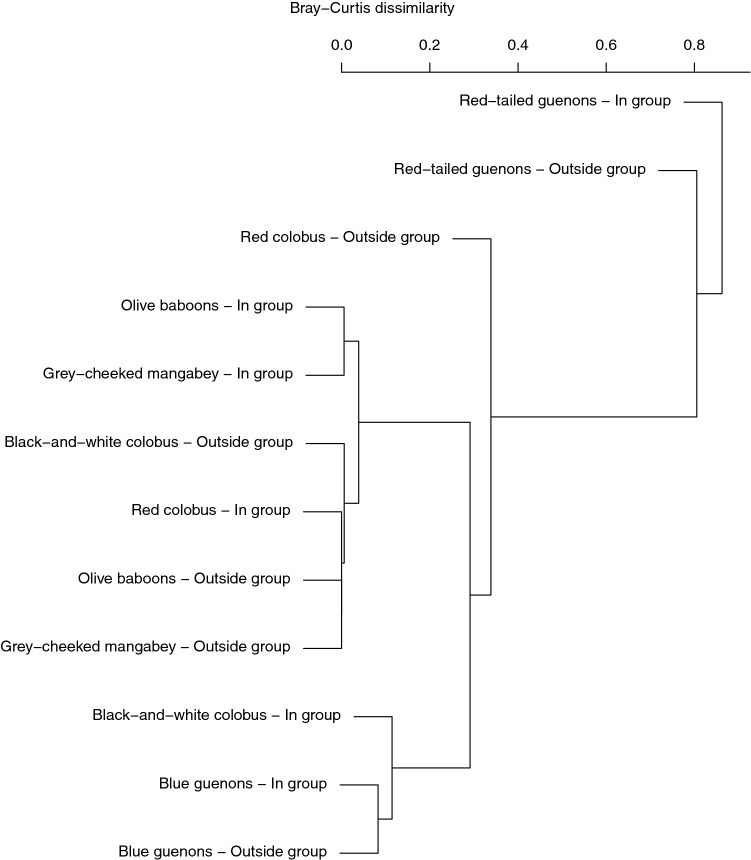
Hierarchical clustering (using the Bray–Curtis distance matrix and the UPGMA algorithm) of fly species community composition inside and outside different primate species. More similar communities cluster more closely with one another.

The fly community in Kibale consisted of flies belonging to the families Calliphoridae, Sarcophagidae, and Muscidae (Table S1); within the Calliphoridae, flies belonging to the species *Chrysomya putoria* were by far the most commonly detected flies, while within the Sarcophagidae it was flies of the species *Sarcophaga haemorrhoidalis*, and in the Muscidae, it was flies of the species *Musca bezzii* (Table S1). Flies captured within and outside the primate groups did not differ in a consistent manner in terms of their species composition (adonis of Bray–Curtis distances: F_1,11_ = 0.00109, R^2^ = 0.001, *P* = 0.957; Fig. 4; Table S1); reads assigned to species belonging to the Calliphoridae ($\bar{x}$_in group_ = 74.1%, *σ*_in group_ = 33.8%, range _in group_ = 11.3–100%; $\bar{x}$_away from group_ = 73.8%, *σ*_away from group_ = 35.6%, range _away from group_ = 9.2–100%) were the most commonly detected inside and away from primate groups. Flies from the Sarcophagidae ($\bar{x}$_in group_ = 11.1%, *σ*_in group_ = 15.0%, range _in group_ = 0.0–36.3%; $\bar{x}$_away from group_ = 21.3%, *σ*_away from group_ = 35.7%, range _away from group_ = 0.0–90.9%), and Muscidae ($\bar{x}$_in group_ = 14.8%, *σ*_in group_ = 36.2%, range _in group_ = 0.0–88.7%; $\bar{x}$_away from group_ = 4.8%, *σ*_away from group_ = 11.9%, range _away from group_ = 0.0–29.2%) were less frequently detected inside and away from primate groups (Table S1).

## Discussion

In Kibale National Park, Uganda, fly densities were generally higher inside primate groups than outside them. When considering data from all primate species together, larger groups of primates were associated with higher fly densities. When examining within-species variation, a positive relationship between group size and fly densities was observed for red colobus and black-and-white colobus. These two species have smaller daily travel distances and home range sizes than the other species studied, likely because of their primarily leaf-based diet (Milton and May 1976). Comparative tests across primates suggest larger daily travel distances and home range sizes are negatively associated with parasite species richness (Nunn et al. 2003). We hypothesize a role of mechanical disease vectors, such as flies, in influencing this relationship if smaller daily travel distances and home range sizes increase vector pressure for social non-human primates more generally. Comparative datasets on the strength of fly associations will allow for rigorous tests of the aspects of host biology (e.g., terrestriality, body mass, home range size, group sizes, group dispersion, defecation rates, diet) that influence fly densities.

Research on vector transmitted parasite risk and its relationship to group size has focused mainly on biological vectors. For example, a comparative study across birds found that transitions from solitary lifestyles to coloniality were associated with increased blood parasite richness; blood parasites were transmitted by different species of vectors suggesting larger aggregations attract not only higher vector numbers, but also a larger diversity of vector species (Tella 2002). Despite this evidence, an experimental study of West Nile virus in passerines showed that group roosting during the non-breeding season protected birds from seroconversion, suggesting a potential benefit of group living with regard to viral infection from mosquitos via the encounter-dilution effect (Krebs et al. 2014). Our results provide further support to the hypothesis that for many primates, flies may also represent a cost of living in larger groups.

The costs of fly associations to primates depend on the flies and pathogens present in an ecosystem. For example, hematophagous flies lead to biting injuries, blood loss, disease transmission, disturbance of rest, and annoyance (Steelman 1976; Dudley and Milton 1990). Flies from the families detected here have been implicated in the mechanical transmission of a diversity of non-human primate infecting pathogens (e.g., *Treponema pallidum pertenue*, the eggs of various parasitic protozoa and helminths, *Bacillus cereus* biovar *anthracis*), suggesting fly associations represent a disease risk (Kumm and Turner 1936; Blackburn et al. 2014; Hoffmann et al. 2017; Gogarten et al. 2019; White et al. 2019). In Kibale, *Chrysomya putoria* (the tropical African latrine blowfly) and *Sarcophaga haemorrhoidalis* were the most commonly detected species detected within primate groups. These species can breed on feces and decaying flesh and have been reported to be mechanical vector of viruses, bacteria, protozoan cysts, and other enteric pathogens, contaminating foods and infecting wounds and thus increasing disease risk (Greenberg 1971; Lindsay et al. 2012). Their specific role in transmitting pathogens in this ecosystem has not yet been explored and this represents an important area of research.

Both of these fly species cause myiasis in humans and livestock, where larvae grow inside the host and feed on its flesh, creating both direct energetic and immunological costs for the host and often leading to secondary infection; in some cases, larvae invade the nervous system, eye, or ear and cause blindness, paralysis, and even death *(*Braverman et al. 1994; Francesconi and Lupi 2012*)*. Myiasis is responsible for extensive human and livestock suffering, but also significant economic losses (Francesconi and Lupi 2012). In primates, the evidence is less clear, though in Panamanian mantled howler monkeys (*Alouatta palliata*) bot fly (*Alouattamyia baeri*) myiasis has been shown to have a major impact on health and mortality, demonstrating the important impact myiasis can have on wild primates (Milton 1996). The risk of myiasis likely represents an additional cost of the fly-primate associations observed in Kibale.

Primates invest considerable resources into insect avoidance. For example, a study of mantled howler monkey slapping of flies and mosquitos suggested monkeys used an average of 1,505 avoidance gestures per day, representing an average energy expenditure of 4.6% of the metabolic costs of living less basal metabolism (Dudley and Milton 1990). Our results suggest fly associations exist for many primate species, raising the question of what defense mechanisms might be used by animals to reduce this vector exposure. Movement patterns, sleeping site selection, or coordination of defecation and movement might reduce fly densities. For example, building a new nest each night may reduce vector exposure for apes (MacKinnon 1974), particularly for vectors that are not able to follow the apes throughout the day. Coordinated defecation and movement could reduce vector presence at food sites if vectors are attracted by feces and then unable to rapidly relocate the group. Neotropical monkeys of the genus *Cebus* rub their fur with arthropods and plants (e.g., the millipede *Orthoporus dorsovittatus*, or plants in the genera *Citrus*, *Clematis*, *Piper*, and *Sloanea*), apparently to deter mosquitos and other insects (Baker 1996; Weldon et al. 2003). Further research is needed to understand whether primates defend themselves against the disease risk posed by fly associations with these or other behaviors.

The finding of similar fly communities within and outside primate groups points toward a random recruitment of primate-associated flies from the environment that creates opportunities for pathogen movement into and out of primate groups. Similarly, the fact that the fly species detected in non-human primate groups are also present in human environments suggests that fly movement between wildlife, humans, and livestock is a possibility. A critical question moving forward will be to determine whether flies move regularly between humans, livestock, and wildlife populations and their potential role in moving pathogens between these populations.

## Supplementary Information

Below is the link to the electronic supplementary material.

Supplementary file1 (XLSX 13 kb)

## Data Availability

COI barcoding reads from this study have been deposited in the European Nucleotide Archive (ENA) at EMBL-EBI under accession number: PRJEB49652.
